# Characterization of the phonological system in low-income Brazilian preschoolers

**DOI:** 10.1590/2317-1782/20232022189

**Published:** 2023-09-15

**Authors:** Maria Helena da Silva, Haydée Fiszbein Wertzner, Charles Haynes, Cíntia Alves Salgado Azoni

**Affiliations:** 1 Programa de Pós-graduação Associado em Fonoaudiologia, Universidade Federal do Rio Grande do Norte - UFRN - Natal (RN), Brasil.; 2 Universidade Federal da Paraíba - UFPB - João Pessoa (PB), Brasil.; 3 Universidade Estadual de Ciências da Saúde de Alagoas - UNCISAL - Maceió (AL), Brasil.; 4 Laboratório LEIA - Linguagem Escrita, Interdisciplinaridade e Aprendizagem, Departamento de Fonoaudiologia, Universidade Federal do Rio Grande do Norte - UFRN - Natal (RN), Brasil.; 5 Departamento de Fisioterapia, Fonoaudiologia e Terapia Ocupacional, Faculdade de Medicina - FM, Universidade de São Paulo USP - São Paulo (SP), Brasil.; 6 Programa de Pós-graduação em Ciências da Reabilitação, Faculdade de Medicina - FM, Universidade de São Paulo USP - São Paulo (SP), Brasil.; 7 Laboratório de Investigação Fonoaudiológica em Fonologia, Faculdade de Medicina - FM, Universidade de São Paulo USP - São Paulo (SP), Brasil.; 8 Department of Communication Sciences and Disorders, School of Health and Rehabilitation Sciences, MGH Institute of Health Professions, Boston (MA), United States of America.

**Keywords:** Speech, Phonology, Child Language, Poverty, Child Development, Speech, Language and Hearing Sciences, Fala, Fonologia, Linguagem Infantil, Pobreza, Desenvolvimento Infantil, Fonoaudiologia

## Abstract

**Purpose:**

The purpose of this study is to characterize the phonological skills of low-income preschool children in a city in the Natal in the Northeast, Brazil.

**Methods:**

The researchers assessed the phonological skills of 90 children (from 5 to 6:11) in early childhood education in three public schools located in regions of social and economic vulnerability. The evaluators used the phonology subtests of the Test of Childhood language (ABFW) children's language test. In addition to performing the standard analysis they examined the following: Phonological Processes (PP), Percentage of Correct Consonants (PCC), Percentage of Correct Consonants Revised (PCC-R), and Process Density Index (PDI). The Spearman's Correlation Coefficient test was used to analyze for correlations among the PCC, PCC-R, and PDI.

**Results:**

According to the cutoff values of children who speak Brazilian Portuguese (BP), adequacy of the PCC and PCC-R values was observed in most participants (PCC: 82 children - 91.1%; PCC-R: 87 children - 94.6%). The processes of liquid simplification (LS), consonant clusters simplification (CCS), final consonant simplification (FCS) were productive of which the CCS (32.2%) and FCS (20%) are still expected for age and LS are not. There was a robust negative correlation between the variables PCC x PDI and PCC-R x PDI.

**Conclusion:**

Most children showed adequate phonological development. Variations were observed in syllabic segments, especially in the coda, which reflect the influence of regional linguistic differences. The evidence obtained regarding the phonological performance of children within this region contributes to a more accurate speech-language diagnosis.

## RESUMO

**Objetivo:**

Caracterizar o sistema fonológico de crianças pré-escolares de baixa renda da cidade de Natal, Nordeste do Brasil.

**Método:**

Foi analisado o nível fonológico de 90 crianças da educação infantil de três escolas públicas localizadas em regiões de vulnerabilidade social. Foi utilizado o instrumento ABFW, nomeação e imitação, por meio da análise tradicional e das medidas de Porcentagem de Consoantes Corretas (PCC), Porcentagem de Consoantes Corretas Revisado (PCC-R) e Índice de Ocorrência de Processos (PDI). Para análise estatística inferencial foi utilizado o teste do Coeficiente de Correlação de Spearman para analisar a correlação entre as variáveis PCC, PCC-R e PDI.

**Resultados:**

De acordo com os valores de corte das crianças que falam Português Brasileiro (PB), a adequação dos valores da PCC e PCC-R foi observada na maioria dos participantes (PCC: 82 crianças - 91,1%; PCC R: 87 crianças - 94,6%). Os processos de simplificação de líquidas (SL), simplificação de encontros consonantais (SEC), simplificação de consoante final (SCF) foram produtivos dos quais o CCS (32,2%) e FCS (20%) ainda são esperados para a idade e SL não. Houve correlação negativa muito forte entre as variáveis PCC x PDI e PCC-R x PDI.

**Conclusão:**

A maioria das crianças apresentou desenvolvimento fonológico adequado. Foram observadas variações nos segmentos silábicos, principalmente na coda, que refletem a influência das diferenças linguísticas regionais. As evidências obtidas sobre o desempenho fonológico das crianças dessa região contribuem para um diagnóstico fonoaudiológico mais preciso.

## INTRODUCTION

Understanding the process of typical phonological development in the face of regional and dialectal variations is important for accurate Speech Therapy diagnoses^(1)^.

Brazilian Portuguese (BP) is generally composed of 7 vowels (/ i /, / e /, / ε /, / a /, / ɔ /, / o /, / u /) and 19 consonants distributed in the following classes: plosives ( / p /, / b /, / t /, / d /, / k /, / g /); nasal (/ m /, / n /, / ɲ /); fricatives (/ f /, / v /, / s /, / z /, / ʃ /, / ʒ /) and liquid (/ X /, / ɾ /, / l /, / ʎ /). In addition, from the combinations of plosives + liquid and fricatives + liquid, it is possible to form 12 consonant clusters (complex onset): / pɾ /, / bɾ /, / tɾ /, / dɾ /, / kɾ /, / gɾ /, / pl /, / bl /, / tl /, / gl / / fr /, / fl /^(2,3)^. Acquisition occurs in an orderly manner, in which sound classes develop sequentially, starting with plosives and nasals, followed by fricatives and liquids^(3,4)^.

Throughout typical phonological acquisition, it is natural that so-called “phonological processes” occur, which are repair strategies used by children in order to adapt the production of the target system to their phonological system. These changes can occur with different natural classes of speech sounds, marked prosodic-syllabic-lexical positions or due to similarities at the intra-segmental and/or segmental levels^(5)^. During the maturation of the phonological system, these processes tend to cease, and the child attains the adult speech patterns.

A study carried out with preschool children who spoke BP in the northeastern region of the country indicated that the phonological processes most present for this population were: simplification of the consonant clusters and liquids, syllable reduction, and simplification of final consonant^(6)^. The process of simplifying the consonant clusters has been found in several previous studies with children in the age group corresponding to the final period of the preschool phase, between 5 and 7 years^(6-11)^. Studies carried out with children who speak other languages indicate variation in the age of suppression of phonological processes^(1,12)^.

This acquisition pattern for phonological processes occurs consistently in BP, and tends to disappear as a function of the child's phonological development. It is essential to highlight that linguistic variants can remain and stem from the regional, cultural, and social aspects in which they occur^(13-15)^. In Brazil, several studies on typical phonological acquisition are concentrated in the south and southeast regions of the country^(2,3,10,11)^. Thus, it is necessary to expand current research to the North and Northeast regions, given the linguistic variation previously reported there^(6,13,15)^.

Phonological processes can differ due to regional variation. A study carried out in the Rio Grande do Sul (Brazil) identified strategies of phonological process repair used by children from 1 to 4 years old to acquire obstruents in two different municipalities. The most commonly used strategy in an urban environment was the syllable or segment omission (Example in Portuguese: bicho - [‘bi0]) and in the other municipality was the posteriorization (Example in Portuguese: urso - [‘uʃu])^(16)^. Another study focusing on the northeastern region of the country indicated variations in the omission of the / R / in the coda position (medial and final) between the states of the region^(13)^.

Given that the phonological abilities of children at preschool age support their development of written language^(17,18)^, it should be considered that some children, especially those with low socioeconomic status, may suffer losses during this early period^(18)^. Considering the environment that they live in, this study hypothesizes that low-income children may have developed the phonological system later. It is crucial to understand how children from low-income regions develop their phonological systems, preparing them for literacy learning. Therefore, this study aimed to provide an in-depth characterization of the phonological system of low-income preschool children in a city in the Northeast.

## METHODS

This study was approved by the Human Research Ethics Committee of the University Hospital of a Federal University of Rio Grande do Norte, under number 2,440,976, and followed the recommendations of Council Resolution 466/2012 National Health Service (CNS). This study is observational, cross-sectional, retrospective and descriptive.

The sample consisted of children studying at Level IV of early childhood education in 2017, at the Municipal Centers for Early Childhood Education (CMEIs) in three regions of the Natal city - West, South, and East; aged 5:0 to 6:11 and of both sexes. All guardians of the children signed a Free and Informed Consent Form.

The CMEIs were appointed by the city's Municipal Education Secretariat (SME), and the direction of each CMEI allowed two classes to participate in the research. The indication criteria used by the SME were the CMEI’s availability of resources and infrastructure to carry out the research. The low-income criterion was analyzed by the Basic Education Development Index (IDEB), which indicates the quality of teaching, considering a score below 4 for those with the worst performance. In this case, the children of these schools met these criteria, as they are located in vulnerable regions and public schools. In addition, the Brazil Criteria was considered, which classifies socioeconomic stratification, which in this case, these children fit in D-E.

All children in the chosen age group were evaluated regarding the phonological system but excluded one who has been diagnosed with neurodevelopmental disorders, genetic syndromes, visual, hearing, or intellectual disabilities or with disorders identified in the teacher's report but who have not yet been diagnosed, and those who did not use oral language as a form of communication were excluded. It is important to note that an audiological evaluation was not performed due to children’s lack of access to the available service; however, children with previous diagnoses or signs of disorders reported by parents in the interview, observation of difficulty in responses during the evaluation of information from teachers about any different behavior or difficulties to answer when called in the classroom.

**Figure 1:**
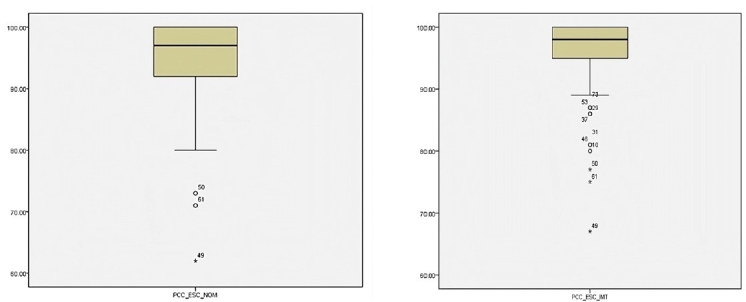
Bloxpot of the percentage of consonants correct in the appointment (a) and percentage of consonants correct in imitation (b) with outliers*

This research was performed by collecting evaluations of 93 children contained in a database that is part of a longitudinal research project at a university in the country's northeastern region, however, three male participants, from the CMEI in the southern zone, one aged between five years and five years and 11 months and two aged between six years and six years and 11 months were excluded from the sample because they presented phonological productions that differed from the average, which would have significantly impacting the result. As can be seen in Figure 1, the boxplot with the PCC values of the phonology tests (word imitation and picture naming) of the phonology test of the children's language test in the areas of phonology, vocabulary, fluency, and pragmatics - ABFW (ABFW phonology)^(9)^, the three children were outliers in both tests.

Regarding the location of children in Natal distributed in the CMEIs, 39 children (43.3%) are from the east, 28 (31.1%) from the west, and 23 (25.9%) from the south. Among them, 44 (48.9%) are male, and 46 (51.1%) are female, aged 5:0 to 6:11.

Thus, the assessments of 90 preschool children in level IV of early childhood education were selected from the current research project's database. The assessment was conducted in a single session and was recorded for later analysis.

These children’s phonological system was analyzed using the following subtests of the ABFW:

- Phonology Test (Part A) of the ABFW - Child Language Test^(9)^: The phonology part (Part A) of the ABFW protocol consists of 2 tests (word imitation and picture naming), in which the phonemes of Brazilian Portuguese can be evaluated in the initial and final positions, the phonemes / h / and / S / in the position of medial and final coda, as well as consonant clusters. This test is suitable for children aged 2 to 12 years. The imitation test has 39 words expressed orally for children. In it, the examiner asks the child to repeat the said word. If the child does not repeat or does so unintelligibly, the examiner requests that he repeat the word at the end of the subtest. The productions must be registered in an appropriate form for phonetic transcription. The picture naming test consists of 34 words depicted in figures presented one by one by the examiner, who asks the child to name the figure. If the child does not know the figure's name, the examiner must state the name, show five figures from the test and then return to the unnamed item. The answers are recorded in an appropriate form for phonetic transcription. When the child does not name the word or does it inappropriately, the examiner records the child’s response. The analysis of the test responses can occur both by the phonetic inventory (standard analysis, in which the correct answers, omissions, substitutions, additions, and distortions are registered) and by the phonological processes (this analysis verifies which rules of Brazilian Portuguese the child is simplifying).

From this response set, the Percentage of Consonants Correct - PCC^(19)^, Percentage of Consonants Correct Revised- PCC-R^(20)^, Process Density Index - PDI^(21)^; as well as phonological processes and their productivity (productive processes with a score above 25% - test reference value) are determined. Thus, in the phonological analysis, traditional phonological processes, PCC, PCC-R, and PDI analysis were used in both the picture naming and word imitation subtests. The PCC is one of the most used analyses to evaluate the production of consonants in a child. It measures the percentage of correctly produced consonants about the total number of consonants present in the speech sample. The PCC-R, on the other hand, takes into account the distortions present in the child's speech and review to assess whether the production of consonants is correct or not.

### Statistical analysis

The data were recorded in a Microsoft Excel database to support the descriptive and inferential statistical analyses. For statistical analysis, SPSS Statistics version 20.0 software was used. The descriptive analysis of categorical variables was performed using absolute (n) and relative (%) frequencies and quantitative variables, using averages, standard deviation, and minimum and maximum values. The normality test considering PCC and PCC-R as dependent variables was performed. The normality of the sample distribution was analyzed using the Kolmogorov Smirnov test since the sample size is less than 100, as shown in Table 1. After this analysis, non-parametric tests were used for inferential analyses.

**Table 1:** Analysis by sample distribution normality according the percentage of consonants correct

**Tests of Normality**				
	Kolmogorov-Smirnov		Shapiro-Wilk	
	N	p	n	p
PCC_ESC_NOM	90	.000	90	.000
PCC-R_ESC_NOM	90	.000	90	.000
PCC_ESC_IMT	90	.000	90	.000
PCC-R_ESC_IMT	90	.000	90	.000

**Caption:** PCC_ESC_NOM: Percentage of Consonants Correct in Naming; PCC-R_ESC_NOM: Percentage of Correct Consonants Revised in Naming; PCC_ESC_IMT: Percentage of Consonants Correct in Imitation; PCC-R_ESC_IMT: Percentage of Correct Consonants Revised in Imitation; n: number of subjects in the sample; p: significant level

The Spearman's correlation coefficient test was used to analyze correlations among the averages of the variables PCC, PCC-R, and PDI. The level of statistical significance adopted for all inferential analysis tests was p≤.05. The magnitude of Spearman's Correlation Coefficient (r) was classified as follows: *negligible* (less than or equal to 0.3), *weak* (between 0.4 and 0.5), *moderate* (between 0.6 and 0.7), *strong* (between 0.8 and 0.9), and *very strong* (greater than 0.91).

## RESULTS

In the phonological analysis, traditional phonological processes, PCC, PCC-R, and PDI analysis were considered in both subtests, picture naming and word imitation, as evaluation criteria. In the standard analysis, the indices of omissions, substitutions, and distortions performed by the children were observed. In the picture naming subtest, it was found that 47 children (52.2%) performed omissions, 49 children (54.4%) substitutions, and nine children (10%) distortions. When analyzing the phonological processes in the picture naming subtest (Table 2), 68 children (73.1%) presented phonological processes, of which 39 (43.3%) displayed at least one productive process.

**Table 2:** Distribution of the number of phonological processes in the naming according the number of processes and production processes

**NP**	**n**	**%**
0	22	24.4
1	17	18.9
2	25	27.8
3	18	20.0
4	6	6.7
5	2	2.2
**NPP**	**n**	**%**
0	51	56.7
1	26	28.9
2	10	11.1
3	3	3.3

**Caption:** NP: number of processes; NPP: number of production processes; n: number of subjects in the sample; %: percentage of subjects in the sample.

The productive phonological processes (PPF) that occurred most frequently in the picture naming were: simplification of the final consonant - 29 (32.2%) (tãboh → tãbo) and simplification of the consonant cluster - 18 (20%) (livru → livu). The distribution of phonological processes in this test can be seen in Table 3.

**Table 3:** Distribution of phonological processes and productive phonological processes in naming

**PP**	**n**	**%**
Syllable Reduction	2	2.2
Fricative Plosivation	1	1.1
Liquid simplification	14	15.6
Consonant Harmony	1	1.1
Palatal fronting	1	1.1
Velar fronting	1	1.1
Simplification of consonant clusters	35	38.9
Final consonant simplification	52	57.8
Plosives deafening	2	2.2
Fricatives deafening	4	4.4
**PPP**	**n**	**%**
Liquid simplification	5	5.6
Simplification of consonant clusters	18	20
Final consonant simplification	29	32.2

**Caption:** PP: phonological processes; PPP: productive phonological processes; n: number of subjects in the sample; %: percentage of subjects in the sample

In the imitation subtest responses, 46 children (51.1%) performed omissions, 27 children (30%) substitutions, and seven children (7.8%) distortions. As for phonological processes in imitation, 62 children (68.9%) had phonological processes; among them, 40 (44.4%) had productive processes (Table 4).

**Table 4:** Distribution of the number of phonological processes and production processes in imitation

**NP**	**N**	**%**
0	28	31.1
1	25	27.8
2	21	23.3
3	10	11.1
4	4	4.4
5	1	1.1
6	1	1.1
**NPP**	**N**	**%**
0	50	55.6
1	31	34.4
2	8	8.9
3	1	1.1

**Caption:** NP: number of processes; NPP: number of production processes; n: number of subjects in the sample; %: percentage of subjects in the sample

The productive phonological processes that occurred most frequently in the imitation subtest were the simplification of the consonant cluster (Example in Portuguese: [pratu - patu]) - 18.9%, followed by the simplification of the final consonant (Example in Portuguese: [tãboh - tãbo]) - 4.4%. Table 5 shows the distribution of phonological processes (productive and non-productive).

**Table 5:** Distribution of phonological processes and productive phonological processes in imitation

**PP**	**N**	**%**
Consonant Harmony	7	7.8
Velar posteriorization	1	1.1
Palatal fronting	2	2.2
Fronting of velar	2	2.2
Liquid simplification	6	6.7
Syllable reduction	1	1.1
Simplification of consonant clusters	33	36.7
Final consonant simplification	29	32.2
Deafening plosives	2	2.2
Deafening of fricatives	2	2.2
**PPP**	**N**	**%**
Simplification of consonant clusters	17	18.9
Final consonant simplification	4	4.4
Liquid simplification	2	2.2

**Caption:** PP: phonological processes; PPP: productive phonological processes; n: number of subjects in the sample; %: percentage of subjects in the sample

When analyzing the responses to the two subtests (picture naming and word imitation), the processes of liquid simplification (LS) (Example in Portuguese: [ ʒirafa - ʒilafa]), consonant cluster simplification (CCS) (Example in Portuguese: [pratu - patu]), and final consonant simplification (FCS) (Ex.: [tãboh - tãbo]) were productive in both. It is important to highlight that among the three processes found, only the liquid simplification is not expected for the age group studied.

As for PCC, the average obtained in the picture naming test was 95.2% (SD = ± 5.3) with a minimum value of 80%, and a maximum of 100%, and PCC-R presented an average of 95.5% (SD = ± 4.8) with minimum values of 80% and maximum of 100%. According to the classification of severity of the PCC^(19)^, there was a variation of degree between mild (82 children, 91.1%) and moderate (8 children, 8.9%, of which three were five years old and the others were older).

In the imitation test, the average PCC was 96.3% (SD = ± 4.7), and PCC-R with an average of 96.6% (SD = ± 4.3), with a minimum value of 80% and a maximum of 100% for both. According to the PCC severity classification, 87 children (94.6%) were classified as mild and three children (5.4%) as mild-moderate. There was slight variation between the scores of the PCC and the PCC-R in the same test (both in the picture naming and in the word imitation), as well as between the tests (picture naming x word imitation).

As for the PDI, the average obtained in the picture naming test was 0.09 (SD = ± 0.12) and presented a minimum value of 0.00 and a maximum of 0.55; in imitation, the average was 0.06 (SD = ± 0.10) and a minimum value of 0.00 and a maximum of 0.51.

The correlation between PCC x PDI and PCC-R x PDI was considered very strong in both subtests (picture naming and word imitation) according to the analysis values of the Spearman's Correlation Coefficient (r> 0.91). In the picture naming test, the correlations between PCC x PDI and PCC-R x PDI showed r = -0.96. In the word imitation test, the correlation coefficient between PCC x PDI was - 0.93, and between PCC-R x PDI was -0.94.

## VARIATION ANALYSES

Through the speech sample, it was also possible to obtain relevant data regarding dialectal variations of these children. The leading segment, which showed variations, was the syllabic coda. All children performed the dialectal variant replacing the phoneme / ʃ / with the phoneme / s / (ex: [pasta] → /paʃta/) in the picture naming and word imitation conditions.

Still, there was an omission of the /h/ phoneme (example in Portuguese: [cohtina] → [cotina]) or replacement of the /R/ phoneme by the semivowel /w/ (example in Portuguese: [gahfo] → [gawfu]). It is worth noting that there were differences in the variations of the /h/ in coda medial between the regions evaluated. It was observed that in the medial coda, seven children (25% from the CMEI of the western part) underwent /h/ substitution, and four children (14,2%) performed the omission /h/ → /Ø/ on the picture naming task; 11 children (28.2% at CMEI in the eastern region) underwent /h/ replacement and 18 children (46.1% at CMEI in the eastern region) completed the omission /h/ → Ø on picture naming and; in CMEI in the south zone, only one child (4.3%) showed /h/ replacement and ten children (43.4%) omission /h/ → /Ø/. Thus, it is possible to verify that 19 children (21.1%) substituted the phoneme /h/ in the medial coda position and 32 children (35.5%) omitted the phoneme /h/ in the medial coda position. The omission of the /h/ in the final coda was observed in eight children (8.8%) from the CMEI in the western region, nine children (10%) in the southern area and 20 children (22.2%) in the eastern part. The data indicated that 37 children (41.1%) omitted the phoneme h in the final coda. In the CMEI of the South area, five children used the allophone [tʃ] (21.7%) and four children used the allophone [dʒ] (17.3%). In CMEI East, two children produced the allophone [tʃ/] (5.1%) and one child produced the allophone [dʒ] (2.5%); in West area no child produced the allophones. Thus, it was possible to observe that 7 children of the total sample (7.7%) produced the allophone [tʃ] and 5 children (5.5%) the allophone [dʒ], being a higher prevalence in the South region of Natal. Additional variations were evident in the vowels. In the CMEI of the West, five children (17.8% of this local sample) were submitted to the substitution of the word for “lettuce” ([ lettuce ] / owfasi /); the same occurred with three children (13%) in the south and 12 children in the east (30.7% of this local sample), which represents 22.2% of the total sample - 20 children. Another aspect observed is that 15 children from the south zone (65.2% of the local sample), 13 from the West area (46.4% of the local sample), and 36 from the East area (92.3% of the local sample) omitted the semivowel (ex: [ scissors ] / tizora /), in this way it is possible to observe that 64 children in the sample presented the same variant, which represents 71.1% of the sample, probably a cultural vocabulary difference in both.

## DISCUSSION

This study showed evidence of unique characteristics of the phonological acquisition of children living in a state in the Northeast of Brazil. On the other hand, it indicated many similarities with the phonological descriptions from studies of children from other regions of the country.

In particular our data indicate that the phonological processes of FCS and CCS present in most of this study’s children’s speech are also found in BP speakers from other regions in the same age groups^(6,9,10)^.

A study carried out with children between typically developing seven and eight years old using the ABFW instrument^(9)^, indicated the same phonological processes^(22)^ expected for the age groups in the current study (SCF e SEC).

It is essential to highlight those five children in our sample (5.6%) showed liquid simplification, which was not expected for their age according to the instrument used^(7)^, with signs of phonological disorder^(10,23)^.

The averages obtained between the PCC and PCC-R measurements were similar (PCC picture naming 94.3% and word imitation 95.5% / PCC-R picture naming 94.6% and word imitation 95.8%). A previous study carried out with children between 5 and 7:11 years old sought to obtain cutoff points for the PCC-R for BP speakers with typical and nontypical development^(24)^. In that research, the phonology test of the ABFW test was also used as an evaluation instrument^(9)^. According to the study, the cutoff values were obtained to determine whether the child had a phonological disorder of 93.4% in the picture naming test and for the word imitation test, two cutoff values were found depending on age, 91% for the age group ≤6: 5 years and 93.9% for the age group> 6: 5 years. As it is possible to observe, the values obtained in this research are above the cutoff point for both subtests, demonstrating that phonological development of most of children in the current study is typical.

As for the PDI, an average of 0.11 and 0.81 in the imitation test was obtained, and there was a solid correlation between the PCC x PDI and PCC-R x PDI. It is essential to highlight that this correlation is negative, indicating that when one value increases, the other decreases, which is expected given they are inversely proportional measures: the higher the consonant success rate, the lower the number of processes used. These results are in agreement with the previous Brazilian literature^(25,26)^.

There were variations in the coda in the phonological analysis, such as replacement or deletion of the phoneme / R /. The possible linguistic variation can justify this behavior of the coda since several studies carried out in different Brazilian regions also point out variation in this segment^(13,27,28)^. A study reported the deletion of / R / in the position of the capital city of the northeast region of the country^(13)^ through the speech sample of the Atlas Linguistic Project of Brazil (ALiB Project). Through this analysis, it was possible to observe that the process is widespread in verbs (V) and less frequent in non-verbs (NV) in the city studied (V: 96%, NV: 71%).

The dialectal variant replacing the phoneme / ʃ / with the phoneme / s / in the medial coda occurred in all children in the sample. A study^(29)^ carried out in the municipality of São José de Mipibu in the state of Rio Grande do Norte found that palatalization occurred in the coda, primarily because of the consonants / t / and / d /. There was variation in the substitution and deletion of the / R / in coda between the regions studied, which was also observed in the vowel / a /. This divergent behavior may be due to unique aspects of each region, including its socio-historical background^(13)^. The low level of social and economic development may not be determinant to increase the risk of speech disorders^(30)^. This variation between regions was also observed in the allophones [tʃ] and [dʒ] since they were present only in the children of the CMEIs in the south and east of the city and absent in the west. A relevant fact is that the CMEIs in the south and east are located close to tourist beaches, which may allow children in these locations to have a greater contact with other cultures, especially in the summer, when the families of these children go in search of work.

The present study allowed us to determine the phonological development typical of preschool children of low socioeconomic levels in a city in the Northeast of Brazil. There are critical linguistic variations, which must be known and considered in phonological analyses. Thus, this study’s findings should reduce errors in possible diagnoses of speech sound disorders, and can help optimize speech diagnosis and treatment essential for at-risk children’s transition from oral language to learning to read and write.

## CONCLUSION

In this study, data are reported that clarify the phonological development of low-income preschool children from public schools in a city in the Northeast.

Most children presented adequate phonological development, PCC and PCC-R within the expected age group, and PDI, with few phonological processes that are not expected for children in the age range studied. Among the few developmentally atypical phonological processes observed, LS and PPF were productive.

Variations were also observed in syllabic segments, especially in the coda, which demonstrates the influence of regional linguistic differences. These data show the importance of knowing and considering linguistic variations in phonological analyses and thus avoiding errors in possible diagnoses of speech sound disorders.

No significant differences were found in the phonological development of these low-income children. Still, it was possible to observe essential variations characteristic of the region of Natal, State Rio Grande do Norte, Brazil that may reflect adult dialectal differences in vocabulary pronunciation.

This study was carried out exclusively with children from public schools and of low socioeconomic level. Thus, we suggest further research to compare the phonological development between high and low-income children in this region to better understand the impact of socioeconomic factors on the variations described here. Future research might include cross-sections of younger children to allow mapping of developmental trajectories.
